# Protecting Tumors by Preventing Human Papilloma Virus Antigen Presentation: Insights from Emerging Bioinformatics Algorithms

**DOI:** 10.3390/cancers11101543

**Published:** 2019-10-12

**Authors:** Elizabeth Gensterblum-Miller, J. Chad Brenner

**Affiliations:** 1 Program in Cellular and Molecular Biology, University of Michigan, Ann Arbor, MI 48109, USA; gensterb@umich.edu; 2 Department of Otolaryngology–Head and Neck Surgery, University of Michigan, Ann Arbor, MI 48109, USA; 3 Department of Pharmacology, University of Michigan, Ann Arbor, MI 48109, USA; 4 Rogel Cancer Center, University of Michigan Medical School, Ann Arbor, MI 48109, USA

**Keywords:** HNSCC, human papilloma virus, neoantigen bioinformatics

## Abstract

Recent developments in bioinformatics technologies have led to advances in our understanding of how oncogenic viruses such as the human papilloma virus drive cancer progression and evade the host immune system. Here, we focus our review on understanding how these emerging bioinformatics technologies influence our understanding of how human papilloma virus (HPV) drives immune escape in cancers of the head and neck, and how these new informatics approaches may be generally applicable to other virally driven cancers. Indeed, these tools enable researchers to put existing data from genome wide association studies, in which high risk alleles have been identified, in the context of our current understanding of cellular processes regulating neoantigen presentation. In the future, these new bioinformatics approaches are highly likely to influence precision medicine-based decision making for the use of immunotherapies in virally driven cancers.

## 1. Introduction

Human papilloma virus (HPV) has garnered public health attention, as HPV infection has been associated with 99.7% of all cases of cervical cancer [1]. However, HPV is also highly associated with head and neck squamous cell carcinoma (HNSCC). HNSCC affects epithelial cells in the region of the mouth and throat spanning the nasal cavity and oral cavity, through the larynx [2]. The region of the head and neck that is most commonly infected by HPV is the oropharynx. In meta-analysis, approximately 20% of all HNSCC cases are HPV-positive, and the overall incidence of HNSCC in young patients continues to rise [3,4]. In the oropharynx, however, 40–60% of all cancers are associated with HPV infection [5] and the proportion of HPV-related HNSCC cases in the United States is increasing, which is thought to be due to changes in both sexual behaviors and smoking habits [6]. Importantly, however, the molecular mechanisms by which HPV infected cells evade the host immune system have only recently begun to come into the limelight, which is largely due to the recent emergence of novel bioinformatics techniques.

Over 100 HPV strains have been found in humans; however, only a few strains have been associated with an increased cancer risk, which are termed high-risk strains [7]. The most frequent of these types is HPV-16, which is associated with 80–90% of all HPV-positive HNSCC cases [5,8]. In the oral mucosa of cancer-free women, infections of low-risk HPV strains are quickly cleared, while infections of high-risk strains, such as HPV-16, continue to linger [9]. Therefore, the factors utilized by high-risk HPV to circumvent efficient immune clearance are thought to contribute to the oncogenic activity of high-risk HPV types.

Several potential mechanisms may contribute to HPV-driven immune evasion. For example, while HPV-positive HNSCC is characterized by decreased presentation of the major histocompatibility complex, class I (MHC class I) on the cell surface, the molecular mechanisms leading to MHC class 1 suppression have only recently begun to emerge [10]. The dysregulation of MHC class I presentation has a profound impact on the T cell-mediated anti-tumor immune response, as MHC class I expression is directly correlated with cytotoxic T cell and natural killer cell infiltration in solid tumors [11,12]. HPV-positive tumors are associated with increased cytotoxic T lymphocyte infiltration [13]. HPV-positive HNSCC tumors also have an increased cytotoxic T lymphocyte-mediated anti-tumor immune response compared to HPV-negative tumors; however, this immune response is negatively correlated with expression of the HPV E7 protein [14,15]. HPV-positive tumors are also associated with other immune changes that are not seen in HPV-negative tumors [16,17]. For example, B cells are less active in HPV-positive HNSCC tumors, and, interestingly, while B cell infiltration is increased in HPV-positive tumors, B cell-mediated antibody production is decreased [18]. HPV positivity is associated with decreased activity in multiple immune cell types. Therefore, recovery of the anti-HPV immune response could improve anti-tumor MHC class I mediated immune response.

Recovery of MHC class I presentation may be poised as an ideal target for immunotherapy for HPV driven cancers, as this increased immune activity could improve tumor clearance. However, previous studies have been limited by the genomic complexity of the MHC class I region. As bioinformatics and sequencing techniques tailored to the MHC genetic locus have been recently developed and improved, and as publicly available MHC class I variation data has expanded over time, these limitations have decreased. Therefore, this review focuses on understanding how recently developed tools and resources impact our understanding of the mechanisms by which high risk HPV drives cancer development and impacts MHC class I presentation.

## 2. Normal MHC Class I Activity 

The MHC class I, which in humans is occasionally also referred to as the human leukocyte antigen (HLA), presents antigens on the surface of normal nucleated cells [19]. The closely related complex MHC class II is involved in antigen-presenting immune cells, and is beyond the scope of this review. The MHC class I presents antigens generated from either endogenous or exogenous peptides on the surface of the cell. When exogenous antigens are presented, such as antigens generated from viral proteins, or neoantigens generated from mutated proteins, the immune system mounts a cytotoxic T lymphocyte and natural killer cell mediated immune response [19]. This was first described in the context of viral infection, where it was demonstrated that antigens generated from viral proteins would trigger an immune response, but only when they are presented by the MHC class I [20]. The MHC class I contains a heavy chain and a light chain; the light chain is transcribed by the *B2M* gene, while the heavy chain is transcribed from either the *HLAA, HLAB,* or *HLAC* genes [19].

Once expressed, MHC class I resides in the endoplasmic reticulum, where small antigens associate with MHC class I. The MHC-antigen complex is subsequently shuttled into the Golgi apparatus, and then continues to the cell surface [21]. At the cell surface, the MHC class I interacts with either the T cell receptor presented by cytotoxic T lymphocytes, or the killer immunoglobulin-like receptor on natural killer cells. If the lymphocyte or natural killer cell recognizes the antigen as exogenous, and therefore potentially pathogenic, the cell will then mount an immune response targeting the exogenous antigen [14]. In a normal cell, T lymphocytes will interact with viral antigens, killing the host cell. Therefore, downregulation of the MHC class I is one strategy by which virus-infected cells can avoid immune attack. In the case of oncogenic viruses, this could cause decreased efficacy of the anti-tumor immune response.

## 3. Neoantigen Identification Using Bioinformatics Methods

Recent advances in bioinformatics techniques have shed light on interactions between various HPV types and neoantigen presentation. The considerations for neoantigen prediction software have been previously reviewed [22,23,24,25,26,27]. Many neoantigen prediction pipelines are available, using a variety of prediction methods (Table 1). The most common method of neoantigen prediction, used in programs such as NetMHC, focuses on modeling the interaction between a predicted neoantigen and the MHC class I molecule [28]. This method uses a neural network to predict the binding affinity of putative neoantigens, based on the HLA allelotype of the sample. Alternative methods include predicting location of peptide cleavage, and interaction with TAP proteins [29,30,31]. Recently developed programs, including NeoPredPipe and NetTepi, use both MHC class I binding affinity and T cell receptor affinity, which leads to improved prediction accuracy [32,33]. In addition, multiple pipelines have been developed that integrate each step of neoantigen prediction into a single workflow, including TIminer, CloudNEO, and pVAC-seq [34,35,36]. Some of these pipelines are specialized to identify neoantigens from specific types of mutations. For example, ScanNEO identifies neoantigens caused by insertion and deletion mutations, while INTEGRATE-Neo was developed to identify neoantigens caused by gene fusions [37,38].

However, each prediction technique has limited efficacy, and predicted neoantigens are not reliably detected in vivo [39]. This is largely because most programs perform neoantigen predictions based on limited aspects of the neoantigen presentation pathway. For example, the widely used prediction program NetMHC predicts neoantigens based on binding affinity to the MHC class I, but does not include neoantigen interaction with T cell receptors, RNA expression of putative neoantigens, or interactions with the neoantigen loading machinery. Programs that integrate multiple neoantigen prediction techniques, such as NeoPredPipe and NetTepi, may increase stringency of the prediction. However, additional pipelines are needed, which can improve prediction accuracy by incorporating additional neoantigen loading pathways into the neoantigen prediction algorithm. Therefore, in vivo validation of predicted neoantigens is necessary. In addition, it has been previously noted that because less binding data is available for rarer HLA haplotypes, often neoantigen prediction for these haplotypes is less accurate [23]. This problem is the motivation for programs such as NetMHCcons, which uses binding information from highly similar peptides to inform the binding patterns of less common haplotypes, which results in a modest increase in prediction accuracy [40]. Recent advances have been made to increase the accuracy of neoantigen prediction tools, though additional methods are still needed.

## 4. Neoantigens in HNSCC

In addition to presenting viral antigens at the cell surface, the MHC class I proteins also present tumor neoantigens. These neoantigens are typically an eight to eleven amino acid peptide sequence that are generated from proteins containing either missense mutations or alternative splicing events [23]. These then undergo antigen loading onto the MHC class I, via similar pathways used in viral antigen loading. In many cancers, identification of neoantigens has attractive therapeutic implications because the presentation of these neoantigens can elicit an anti-tumor immune response. In addition, vaccines that target these neoantigens are an attractive immunotherapy, and have been shown improve T cell mediated immune response in melanoma patients [41,42]. Clinical trials have been started for neoantigen vaccines in many additional cancer types, including glioma, bladder carcinoma, non-small cell lung cancer, pancreatic cancer, and triple negative breast cancer, (summarized in Chu et al. [43]).

Neoantigen identification in HNSCC is an attractive target because HNSCC tumors have a moderately high mutational burden in comparison with other cancers [44,45], so a high number of putative neoantigens are possible [46]. Indeed, HNSCC is found to have increased tumor mutational burden, as well as increased predicted neoantigen burden, when compared to other solid tumor types [47]. This study predicts putative neoantigens across a large patient cohort using the prediction program NetMHCpan, identifying neoantigens that are predicted to bind to the MHC class I with high affinity. However, studies such as this one are limited by the prediction methods currently available. Programs such as NetMHCpan use a neural network to predict antigen binding efficiency to the MHC class I, but do not predict other aspects of neoantigen processing, such as peptide cleavage, and neoantigen-T cell interaction. The specific putative neoantigens identified have not been shown to be biologically relevant, as it is not known if they will activate T cells, triggering a T cell-mediated anti-tumor immune response. Only a small fraction of these predicted neoantigens will actually play a role in immune regulation, so the actual biological role of specific neoantigens must be validated. One recent study identified neoantigens in ten HNSCC patients. Neoantigens were predicted using a similar pipeline as the previous study, but then each neoantigen was validated by testing each neoantigen’s ability to activate patient CD8+ T cells [48]. While 15 predicted neoantigens were identified, this study found that only two are able to trigger an immune response. Another study identified predicted neoantigens in HNSCC tumor samples, and treated patient T cells with these neoantigen peptides [49]. They then measured T cell activation to determine the immunogenicity of each neoantigen. The authors described one specific neoantigen, the result of a gene fusion, that elicits an immune response, of all predicted neoantigens tested. These results suggest that gene fusion-derived neoantigens cause an increased immune response, compared to missense-derived neoantigens. Each of these studies successfully identify patient-specific neoantigens in HNSCC, which could be useful in future studies developing personalized immunotherapies, including neoantigen vaccines. However, they also illustrate current limitations in predicting the biological relevance of specific neoantigens, which is largely due to the complexity of both neoantigen presentation and neoantigen-mediated T cell activation.

## 5. MHC Class I Genotype May Affect HPV Antigen Presentation

As part of normal MHC function, HPV antigens are generated from translated peptides, then loaded onto the MHC class I antigen binding groove for surface presentation. Individual allelotypes have unique binding affinities to HPV antigens, and, therefore, HPV neoantigen load is also expected to vary between individual patients. In fact, recent studies have shown that the frequency and prognosis of HPV-positive cervical cancers are both associated with specific HLA haplotypes. For example, an association between HLA-B*07 and HPV-positive cervical cancer has been identified in multiple ethnic groups [50,51,52]. This association has been identified in three independent populations, which each perform PCR sequence typing to identify HLA types present in each individual: 42 British cervical cancer patients and 946 healthy controls; 50 cervical cancer patients and 89 healthy controls from the Hubei province in China; and 141 high grade, 202 low grade, and 202 HPV-positive healthy patients in the United States. This led to the hypothesis that specific haplotypes may be more amenable to transition to HPV-driven cancer following HPV infection because certain haplotypes may not present as many HPV peptides on the cell surface.

**Figure 1:**
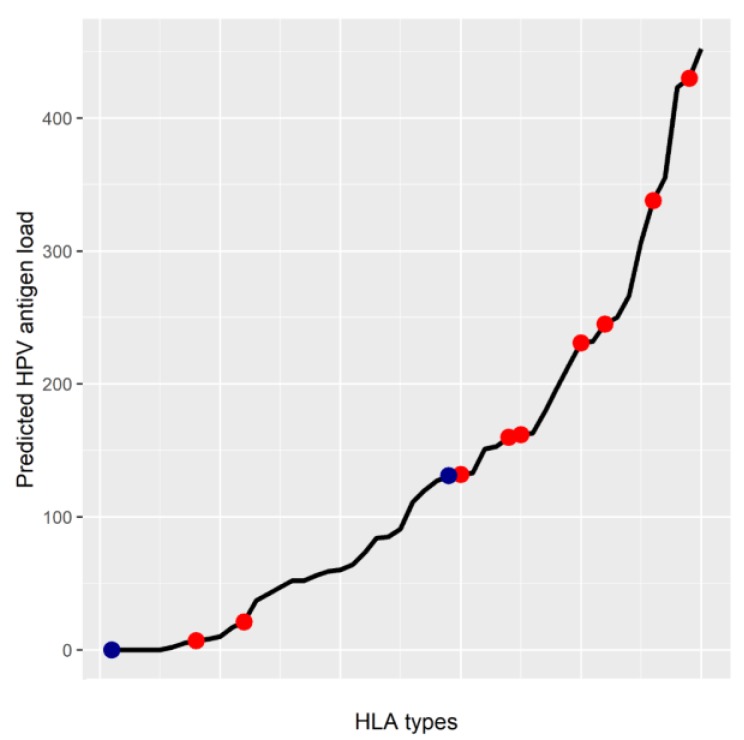
The predicted HPV antigen load of all common MHC class I haplotypes. All possible antigens were generated from the canonical peptide sequences encoded from HPV16, and each antigen’s binding affinity to each HLA haplotype was predicted using NetMHC 4.0 [28]. Predicted HPV antigen load is the sum of all HPV antigens predicted to strongly bind to each MHC class I protein (binding affinity < 500 nM). All MHC haplotype available from the immune epitope database are included in this analysis [53]. Specific HLA alleles are highlighted. Red points correspond to HLA alleles associated with increased risk of cervical cancer, and blue points are protective alleles for cervical cancer [54].

Interestingly, to put the hypotheses generated by these papers in context using recently developed informatics tools, we used the NetMHC 4.0 to define the distribution of HPV peptide neoantigen presentation load across all known HLA haplotypes. Indeed, the predicted HPV16-based antigen load was highly dependent on the HLA allelotype of the infected individual (Figure 1). Surprisingly, however, the number of predicted neoantigens did not necessarily correlate the reported increased risk of HPV-positive cervical cancer (Figure 1) as different high risk haplotypes had a widely varying predicted neoantigen load. This lack of a correlation could be due to many factors. For example, intergenic variants or pathway mutations may play a role in neoantigen presentation that is not described by the HLA haplotype alone. Also, recent research has also shown that HPV oncoproteins also directly regulate MHC expression as described below, as such it is possible that certain HPV haplotypes are also more stringently regulated by HPV oncoproteins. Therefore, additional research is necessary to determine the role of individual mutations, and their effects on HLA regulation.

## 6. The Role of MHC Class I Changes in HNSCC

Early studies have documented that the MHC class I is lost in approximately 50% of HNSCC tumors [55]. Though these studies do not classify HPV status, more recent studies have identified MHC class I loss in both HPV-positive and HPV-negative HNSCC, as HPV-positive tumors had a lower surface level of MHC class I than HPV-negative tumors [10]. This study measured MHC class I surface expression by immunohistochemistry of paraffin-embedded biopsies, using 27 HPV-positive and 68 HPV-negative tumors. There is some debate, however, about the prognostic effects of MHC class I loss [10,56]. Despite these initial data, large-scale studies, as well as meta-analyses, are still necessary to determine the association of MHC class I expression and prognosis. Although the studies completed to date demonstrate that MHC class I loss is detected in HNSCC, regardless of HPV status, the specific mechanism of MHC class I loss in both tumor subtypes remains incompletely understood. Thus, further work is also necessary to determine the processes involved in MHC loss, and the differences between HPV-positive and negative MHC loss.

**Figure 2:**
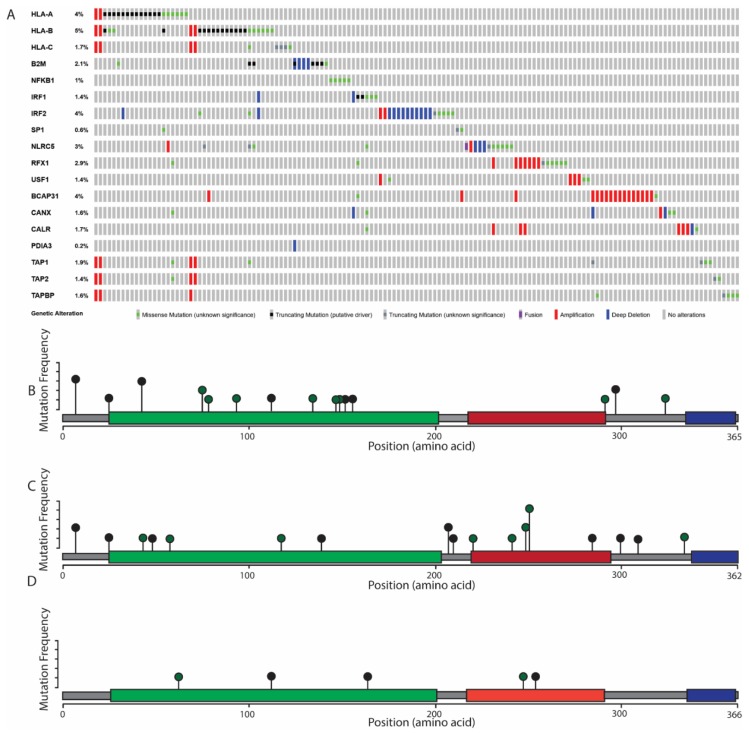

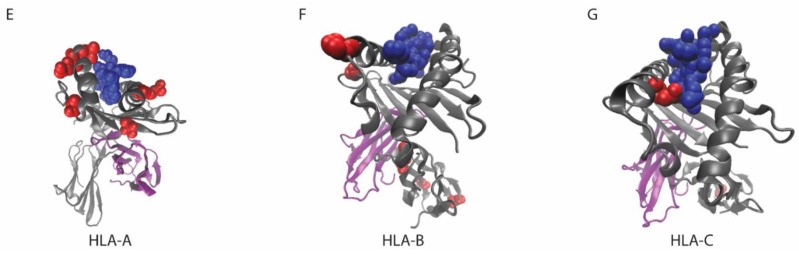
Mutation frequency and localization of MHC class I complex and related proteins. (**A**) mutation frequency of members of the MHC. HLA-A, B, and C, are paralogs of the MHC heavy chain, and β-2 microglobulin (*B2M*) forms the MHC light chain. 8% of HNSCC tumors have a somatic mutation in one or more HLA gene, and 2% have a mutation in *B2M*. 21% of all tumors have a somatic mutation in one or more genes associated with either MHC class I transcriptional regulation or the antigen loading pathway. *NFKB*, *IRF1*/*2*, *SP1*, *NLRC5*, *RFX1*, and *USF1* are each transcription factors that have been shown to interact with the HLA promoter region in various cell types [57,58,59]. The transcription factors that regulate MHC class I have not been described in HNSCC. *BCAP31* is necessary for shuttling the MHC class I from the ER to the GA, and each of the remaining proteins has a role in MHC class I folding and neoantigen loading within the ER [60,61,62,63]. Transcriptional regulation of the MHC class I has been shown to depend on a complex network of cis and trans-acting regulation, so a comprehensive list of transcription factors with a defined role in MHC class I regulation has not been fully determined [64]. (**B**–**D**) Localization of mutations detected in HNSCC patients in HLA-A (**B**), HLA-B (**C**), and HLA-C (**D**). Black dots represent a nonsense mutation; green dots represent a missense mutation. Peptide domains are marked on each MHC class I peptide. Green domain: MHC class I alpha 1 and 2 domains. Red: C1-set domain. Blue: C terminal domain. (**E**–**G**) Location of somatic missense mutations within the HLA protein detected in HNSCC tumors. The HLA protein consists of three α domains: α-1 and -2 contain the antigen binding groove, while the α-3 domain interacts with β-2 microglobulin. Purple: β-2 microglobulin. Grey: HLA heavy chain, including the α-1–3 domains. Blue: neoantigen peptide, located in the antigen binding groove. Red: amino acids that are subject to somatic missense mutation in one or more HNSCC patient. Mutations in HLA-A (**E**) are localized to the antigen binding groove, eight total missense mutations. Mutations in HLA-B (**F**) are present in each α domain, seven total missense mutations. Mutations in HLA-C (**G**) are present in the α-3 domain as well as the antigen binding groove, two total missense mutations. Mutation data retrieved from the TCGA PanCancer Atlas.

Recent sequencing analysis of HNSCC tumors has identified one mechanism of MHC inactivation. Somatic mutations in both HLA proteins and related pathway proteins have been documented in HNSCC. In a large HNSCC patient cohort published by the TCGA cancer atlas (*n* = 515), 9% (45/515 of all HNSCC patients have somatic mutations in the MHC class I heavy chain genes (*HLA-A, HLA-B,* and *HLA-C*), or the MHC class I light chain gene (β-2 microglobulin, *B2M*) (Figure 2). These mutations include nonsense mutations, which lead to loss of the MHC class I, as well as several missense mutations. Of particular note, 61% (11/18) from this cohort have missense mutations found in the antigen binding groove of the MHC class I protein, which are predicted to lead to altered neoantigen binding specificity within the tumor (Figure 2).

In multiple types of cancer, including HNSCC, enrichment of loss-of-function mutations, including missense mutations in the antigen binding groove and nonsense mutations, has been documented [47,65]. Moreover, in multiple cancer types, MHC class I loss-of-function mutations, including mutations in the HLA genes and β-2 micro-globulin, are associated with decreases in markers of CD8+ T cell activity [47], suggesting that the mutations identified in the HNSCC TCGA project as well as in HNSCC cell line panels [66,67] also lead to decreased CD8+ T-cell activity in this tumor type.

In addition to mutations in the MHC class I protein, mutations in the pathways that control transcriptional regulation, neoantigen loading, or protein trafficking of the MHC class I have been found in 21% of HNSCC patients in the TCGA cancer atlas (106/515) (Figure 2). These mutations could putatively affect MHC class I expression or antigen binding, but many have not yet been individually validated. As such, additional studies are needed to generate a comprehensive list of all proteins involved with MHC class I gene expression, especially in squamous tissues, as it has been shown that a wide variety of transcription factors are involved in HLA regulation and that the role of these transcription factors is highly dependent on cell type [64]. Consequently, the role of these neoantigen pathway mutations in MHC class I-mediated processes, such as neoantigen presentation, has not been fully described. Moreover, bioinformatics tools predicting the effect of these pathway mutations on antigen presentation are not currently available. Therefore, additional studies are needed to determine the specific transcription factors that are necessary for HLA transcriptional regulation in HNSCC and to determine the functional role of antigen presentation pathway mutations.

## 7. Considerations for MHC Class I Mutations

Limitations in describing the genetic regulation of the MHC class I are often due to the high density of variants in the MHC genetic region. Additional whole-genome sequencing studies are needed to identify somatic mutations that affect the activity of MHC class I in HNSCC patients. While exonic mutations certainly play a role in the activity of the MHC class I peptide, it has been shown that intergenic mutations and variation play a significant and complex role in the transcriptional regulation of the MHC class I [64]. The role of variation within the MHC genomic region is incompletely understood, which is partially due to unique technical considerations when studying this genomic region.

The MHC genomic region refers to a four megabase portion of chromosome 6 that contains all MHC class I genes, as well as the MHC class II, which is involved in a separate pathway of antigen presentation by immune cells. This region is one of the most genetically complicated regions of the human genome, with a high density of variation between individuals. MHC genetic variation is generalized into named haplotypes for each MHC gene; currently, 13,680 validated MHC class I gene haplotypes have been described worldwide [68]. Moreover, the MHC genomic region is characterized by a complex network of gene regulation, which is highly dependent on MHC genetic variation [64,69]

In HNSCC, the effect of MHC class I genetic variation on anti-cancer immune activity and HPV-MHC interactions has not been described. In addition, transcriptional regulation of MHC class I is highly cell type-specific, so many known mechanisms of MHC class I transcriptional regulation have not been validated in the context of HNSCC [64]. Interesting future directions of study to characterize this regulation include: functionally relevant intergenic somatic mutations, the effect of HLA haplotypes in HNSCC neoantigen presentation, and the transcription factors involved in MHC class I regulation in HNSCC. Before the role of HPV proteins can be fully described in individual patients, the role of intrinsic variation between individuals will have to be considered.

## 8. HPV E7 Inhibits MHC Class I Gene Expression

MHC class I loss in HPV-positive cancers could be directly caused by specific HPV proteins with the MHC genetic locus. Two HPV proteins, E6 and E7, are important oncogenes, and play a role in the development of HNSCC. HPV E6 and E7 are constitutively expressed throughout viral infection [70]. E6 degrades p53, an important tumor suppressor gene critical for cell cycle arrest [71]. Interestingly, in HPV-negative HNSCC tumors, which lack E6 activity, p53 loss of function mutations occur at a much higher frequency than in HPV-positive cases [72]. In addition, E7 interacts with retinoblastoma protein (RB), another tumor suppressor [73]. However, E7 protein also has a significant role in aberrant gene regulation of infected cells, and E7 is directly linked to dysregulation of MHC class I transcription.

In HEK-293 cell lines infected with high-risk HPV strains, MHC mRNA levels are much lower than in samples infected with low-risk HPV strains [74]. Moreover, E7 proteins from high-risk HPV strains are directly associated with MHC class I expression [12]. HPV E7 is reported to bind to, and repress, HLA promoters [75,76]. However, these reports were published before high-throughput techniques revealed a complex network of trans-regulation in the MHC class I genetic region [64]. Therefore, the implications of these promoter binding patterns must be reconsidered in the greater context of complicated regulation that is the hallmark of the MHC region. For example, E7 may interact with distal enhancers, as well as the HLA promoters. Alternatively, proteins or mutations at these distal enhancers may play a role in regulating E7 inhibition of HLA gene expression.

E7 has been shown to interact with the *HLA-A* promoter, and could interact with other promoters and enhancers in the HLA genetic region. While directly interacting with the promoters of HLA genes, E7 could recruit specific epigenetic editor proteins, such as histone deacetylases or DNA methyltransferases, which directly alter the epigenetic patterns at each HLA promoter, which would result in repressed transcription of each HLA gene. E7 has been shown to recruit histone deacetylases, which remove open chromatin marks, reducing the region’s openness to transcription [74]. In addition, transcriptional repression of MHC class I genes can be reversed by treatment with a DNMT1 inhibitor [77]. DNMT1, or DNA methyltransferase 1, maintains DNA methylation patterns, and also produces de novo methylation events. Because HLA transcription increases after DNMT1 inhibition, HLA transcriptional repression could be mediated, at least partially, by DNA methylation. Moreover, this same study describes that after recovery of MHC class I expression by DNMT inhibitor treatment, immunotherapy efficacy improves in a mouse HPV-16-positive tumor model [77]. These promising results demonstrate that recovery of MHC class I presentation is an attractive target for improving immunotherapy treatments.

## 9. The Next Steps of HPV-Targeting Therapies

High-risk strains of HPV have been shown to inhibit MHC class I surface presentation in cervical cancer. Constitutively expressed viral proteins E6 and E7 inhibit expression of MHC class I proteins, while E5 prevents trafficking of the MHC class I to the cell membrane. Decreased MHC class I presentation on the cell surface then interferes with immune attack of the infected cell. However, it remains unclear how this interference with immune response can be rescued in the context of HNSCC. These studies have the potential to improve treatment of HPV, through the identification and inhibition of HPV-specific pathways. For example, increasing expression of the MHC class I by inhibition of HPV proteins E5 or E7 could improve patient outcomes.

Previous studies describing the role of HPV in MHC class I regulation have been successful in proposing specific mechanisms, but they have been limited by the variability of both the MHC protein and the MHC genomic region. These issues have been addressed by recent bioinformatics techniques, including sequence alignment workflows and antigen binding affinity predictions. By implementing these methods, future studies will be able to characterize the effect of HPV within the complex MHC class I regulatory network that has been described. However, new tools are also needed to improve antigen prediction accuracy and to further address limitations caused by MHC genetic variability. Therefore, the use of bioinformatics and sequencing methods developed for working with the MHC may better inform treatment for HPV-positive cancers.

## Figures and Tables

**Table 1:** Bioinformatics methods available for neoantigen prediction.

Program Name	Input Data Type	Summary	Website
CloudNeo	WES or WGS	Integrates neoantigen peptide sequence calling, HLA typing, and peptide-MHC binding affinity predictions	https://github.com/TheJacksonLaboratory/CloudNeo
INTEGRATE-neo	RNA-seq	Integrates gene fusion identification	https://github.com/ChrisMaherLab/INTEGRATE-Neo
NeoPredPipe	variant call set	Integrates putative neoantigen peptide sequence identification and MHC binding affinity prediction	https://github.com/MathOnco/NeoPredPipe
NetChop	Peptide sequences	Predicts peptide cleavage sites	http://www.cbs.dtu.dk/services/NetChop
NetMHC	Peptide sequences, MHC haplotype	Predicts neoantigen binding affinity in an MHC type-dependent manner	http://www.cbs.dtu.dk/services/NetMHC
NetMHCcons	Peptide sequences, MHC DNA sequence	Predicts antigen binding affinities of rare MHC haplotypes	http://www.cbs.dtu.dk/services/NetMHCcons
NetMHCpan	Peptide sequences, MHC haplotype	Similar to NetMHC, with more MHC types included in training data	http://www.cbs.dtu.dk/services/NetMHCpan
NetTepi	Peptide sequences, MHC haplotype	Predicts neoantigen activity by combining peptide-MHC binding affinity and stability, and T cell propensity	http://www.cbs.dtu.dk/services/NetTepi
Pcleavage	Protein sequences	Predicts peptide cleavage sites	http://crdd.osdd.net/raghava/pcleavage
PRED(TAP)	Peptide sequences	Predicts peptide-TAP binding patterns	antigen.i2r.a-star.edu.sg/predTAP (currently unavailable)
pVAC-Seq	WES or WGS and RNA-seq	Combines variant calling and RNA-seq to identify transcribed putative antigens	https://github.com/griffithlab/pVAC-Seq
ScanNeo	RNA-seq	Neoantigen sequence prediction, optimized for indel mutations	https://github.com/ylab-hi/ScanNeo
TIminer	RNA-seq, somatic mutation calling	Integrates RNA-seq and somatic mutations to predict expressed neoantigens	https://icbi.i-med.ac.at/software/timiner/timiner.shtml

Each algorithm described in this review is included in the table. Peptide sequences are typically generated from nonsynonymous coding mutations, identified from variant call sets. WES: whole exome sequencing. WGS: whole genome sequencing. TAP Transporter associated with antigen processing. Indel: Insertion-deletion mutation.

## Abbreviations

HLA	human leukocyte antigen.
B2M	β-2 microglobulin.
NFKB	nuclear factor κ b.
IRF	interferon regulatory factor.
SP	specificity protein.
NLRC	NLR Family CARD Domain Containing.
RFX	Regulator factor X.
USF	upstream transcription factor.
BCAP	B cell receptor associated protein.
CANX	calnexin.
CALR	calreticulin.
PDIA	Protein Disulfide Isomerase Family A.
TAP	Transporter associated with antigen processing.
TAPBP	Tapasin. Mutation data retrieved from the TCGA PanCancer Atlas.

## References

[1] Walboomers J.M., Jacobs M.V., Manos M.M., Bosch F.X., Kummer J.A., Shah K.V., Snijders P.J., Peto J., Meijer C.J., Muñoz N. (1999). Human papillomavirus is a necessary cause of invasive cervical cancer worldwide. J. Pathol..

[2] Michmerhuizen N.L., Birkeland A.C., Bradford C.R., Brenner J.C. (2016). Genetic determinants in head and neck squamous cell carcinoma and their influence on global personalized medicine. Genes Cancer.

[3] Gingerich M.A., Smith J.D., Michmerhuizen N.L., Ludwig M., Devenport S., Matovina C., Brenner C., Chinn S.B. (2018). Comprehensive review of genetic factors contributing to head and neck squamous cell carcinoma development in low-risk, nontraditional patients. Head Neck.

[4] Tillman B.N., Yanik M., Birkeland A.C., Liu C.-J., Hovelson D.H., Cani A.K., Palanisamy N., Carskadon S., Carey T.E., Bradford C.R. (2016). Fibroblast growth factor family aberrations as a putative driver of head and neck squamous cell carcinoma in an epidemiologically low-risk patient as defined by targeted sequencing. Head Neck.

[5] Marur S., D’Souza G., Westra W.H., Forastiere A.A. (2010). HPV-associated head and neck cancer: A virus-related cancer epidemic. Lancet Oncol..

[6] D’Souza G., Fakhry C., Gillison M.L., Kreimer A.R., Viscidi R., Pawlita M., Koch W.M., Westra W.H. (2007). Case–Control Study of Human Papillomavirus and Oropharyngeal Cancer. N. Engl. J. Med..

[7] Braaten K.P., Laufer M.R. (2008). Human Papillomavirus (HPV), HPV-Related Disease, and the HPV Vaccine. Rev. Obstet. Gynecol..

[8] Nulton T.J., Olex A.L., Dozmorov M., Morgan I.M., Windle B. (2017). Analysis of The Cancer Genome Atlas sequencing data reveals novel properties of the human papillomavirus 16 genome in head and neck squamous cell carcinoma. Oncotarget.

[9] Louvanto K., Rautava J., Willberg J., Wideman L., Syrjanen K., Grenman S., Syrjanen S. (2013). Genotype-Specific Incidence and Clearance of Human Papillomavirus in Oral Mucosa of Women: A Six-Year Follow-Up Study. PLoS ONE.

[10] Ou D., Adam J., Garberis I., Blanchard P., Nguyen F., Levy A., Casiraghi O., Gorphe P., Breuskin I., Janot F. (2019). Influence of tumor-associated macrophages and HLA class I expression according to HPV status in head and neck cancer patients receiving chemo/bioradiotherapy. Radiother. Oncol..

[11] Campo M., Graham S., Cortese M., Ashrafi G., Araibi E., Dornan E., Miners K., Nunes C., Man S., Graham S. (2010). HPV-16 E5 down-regulates expression of surface HLA class I and reduces recognition by CD8 T cells. Virology.

[12] Bottley G., Watherston O.G., Hiew Y.L., Norrild B., Cook G.P., Blair G.E. (2008). High-risk human papillomavirus E7 expression reduces cell-surface MHC class I molecules and increases susceptibility to natural killer cells. Oncogene.

[13] Partlová S., Boucek J., Kloudova K., Lukesova E., Zabrodsky M., Grega M., Fučíková J., Truxova I., Tachezy R., Spisek R. (2015). Distinct patterns of intratumoral immune cell infiltrates in patients with HPV-associated compared to non-virally induced head and neck squamous cell carcinoma. OncoImmunology.

[14] Krishna S., Ulrich P., Wilson E., Parikh F., Narang P., Yang S., Read A.K., Kim-Schulze S., Park J.G., Posner M. (2018). Human papilloma virus specific immunogenicity and dysfunction of CD8+ T cells in head and neck cancer. Cancer Res..

[15] Russell S., Angell T., Lechner M., Liebertz D., Correa A., Sinha U., Kokot N., Epstein A. (2013). Immune cell infiltration patterns and survival in head and neck squamous cell carcinoma. Head Neck Oncol..

[16] Hoesli R., Birkeland A.C., Rosko A.J., Issa M., Chow K.L., Michmerhuizen N.L., Mann J.E., Chinn S.B., Shuman A.G., Prince M.E. (2018). Proportion of CD4 and CD8 tumor infiltrating lymphocytes predicts survival in persistent/recurrent laryngeal squamous cell carcinoma. Oral Oncol..

[17] Mann J.E., Smith J.D., Birkeland A.C., Bellile E., Swiecicki P., Mierzwa M., Chinn S.B., Shuman A.G., Malloy K.M., Casper K.A. (2019). Analysis of tumor-infiltrating CD103 resident memory T-cell content in recurrent laryngeal squamous cell carcinoma. Cancer Immunol. Immunother..

[18] Lechner A., Schlößer H.A., Thelen M., Wennhold K., Rothschild S.I., Gilles R., Quaas A., Siefer O.G., Huebbers C.U., Cukuroglu E. (2019). Tumor-associated B cells and humoral immune response in head and neck squamous cell carcinoma. OncoImmunology.

[19] Adiko A.C., Babdor J., Gutiérrez-Martínez E., Guermonprez P., Saveanu L. (2015). Intracellular Transport Routes for MHC I and Their Relevance for Antigen Cross-Presentation. Front. Immunol..

[20] Doherty P.C., Zinkernagel R.M. (1975). Enhanced immunological surveillance in mice heterozygous at the H-2 gene complex. Nature.

[21] Gruener M., Bravo I.G., Momburg F., Alonso A., Tomakidi P. (2007). The E5 protein of the human papillomavirus type 16 down-regulates HLA-I surface expression in calnexin-expressing but not in calnexin-deficient cells. Virol. J..

[22] Hackl H., Charoentong P., Finotello F., Trajanoski Z. (2016). Computational genomics tools for dissecting tumour–immune cell interactions. Nat. Rev. Genet..

[23] Hutchison S., Pritchard A.L. (2018). Identifying neoantigens for use in immunotherapy. Mamm. Genome.

[24] Lee C.-H., Yelensky R., Jooss K., Chan T.A. (2018). Update on Tumor Neoantigens and Their Utility: Why It Is Good to Be Different. Trends Immunol..

[25] Lundegaard C., Hoof I., Lund O., Nielsen M. (2010). State of the art and challenges in sequence based T-cell epitope prediction. Immunome Res..

[26] Soria-Guerra R.E., Nieto-Gomez R., Govea-Alonso D.O., Rosales-Mendoza S. (2015). An overview of bioinformatics tools for epitope prediction: Implications on vaccine development. J. Biomed. Inform..

[27] Snyder A., Chan T.A. (2015). Immunogenic peptide discovery in cancer genomes. Curr. Opin. Genet. Dev..

[28] Andreatta M., Nielsen M. (2016). Gapped sequence alignment using artificial neural networks: Application to the MHC class I system. Bioinformatics.

[29] Nielsen M., Lundegaard C., Lund O., Kesmir C. (2005). The role of the proteasome in generating cytotoxic T-cell epitopes: Insights obtained from improved predictions of proteasomal cleavage. Immunogenetics.

[30] Bhasin M., Raghava G.P.S. (2005). Pcleavage: An SVM based method for prediction of constitutive proteasome and immunoproteasome cleavage sites in antigenic sequences. Nucleic Acids Res..

[31] Zhang G.L., Petrovsky N., Kwoh C.K., August J.T., Brusic V. (2006). PRED (TAP): A system for prediction of peptide binding to the human transporter associated with antigen processing. Immunome Res..

[32] Trolle T., Nielsen M. (2014). NetTepi: An integrated method for the prediction of T cell epitopes. Immunogenetics.

[33] Schenck R.O., Lakatos E., Gatenbee C., Graham T.A., Anderson A.R. (2019). NeoPredPipe: High-throughput neoantigen prediction and recognition potential pipeline. BMC Bioinform..

[34] Hundal J., Carreno B.M., Petti A.A., Linette G.P., Griffith O.L., Mardis E.R., Griffith M. (2016). pVAC-Seq: A genome-guided in silico approach to identifying tumor neoantigens. Genome Med..

[35] Bais P., Namburi S., Gatti D.M., Zhang X., Chuang J.H. (2017). CloudNeo: A cloud pipeline for identifying patient-specific tumor neoantigens. Bioinformatics.

[36] Tappeiner E., Finotello F., Charoentong P., Mayer C., Rieder D., Trajanoski Z. (2017). TIminer: NGS data mining pipeline for cancer immunology and immunotherapy. Bioinformatics.

[37] Wang T.-Y., Wang L., Alam S.K., Hoeppner L.H., Yang R. (2019). ScanNeo: Identifying indel derived neoantigens using RNA-Seq data. Bioinformatics.

[38] Zhang J., Mardis E.R., Maher C.A. (2017). INTEGRATE-neo: A pipeline for personalized gene fusion neoantigen discovery. Bioinformatics.

[39] Chen Y.P., Wang Y.Q., Lv J.W., Li Y.Q., Chua M.L.K., Le Q.T., Lee N., Colevas A.D., Seiwert T., Hayes D.N. (2019). Identification and validation of novel microenvironment-based immune molecular subgroups of head and neck squamous cell carcinoma: Implications for immunotherapy. Ann. Oncol..

[40] Karosiene E., Lundegaard C., Lund O., Nielsen M. (2012). NetMHCcons: A consensus method for the major histocompatibility complex class I predictions. Immunogenetics.

[41] Ott P.A., Hu Z., Keskin D.B., Shukla S.A., Sun J., Bozym D.J., Zhang W., Luoma A., Giobbie-Hurder A., Peter L. (2017). An immunogenic personal neoantigen vaccine for patients with melanoma. Nature.

[42] Sahin U., Derhovanessian E., Miller M., Kloke B.-P., Simon P., Löwer M., Bukur V., Tadmor A.D., Luxemburger U., Schrörs B. (2017). Personalized RNA mutanome vaccines mobilize poly-specific therapeutic immunity against cancer. Nature.

[43] Chu Y., Liu Q., Wei J., Liu B. (2018). Personalized cancer neoantigen vaccines come of age. Theranostics.

[44] Birkeland A.C., Yanik M., Tillman B.N., Scott M.V., Foltin S.K., Mann J.E., Michmerhuizen N.L., Ludwig M.L., Sandelski M.M., Komarck C.M. (2016). Identification of Targetable ERBB2 Aberrations in Head and Neck Squamous Cell Carcinoma. JAMA Otolaryngol. Neck Surg..

[45] Giefing M., Wierzbicka M., Szyfter K., Brenner J., Braakhuis B., Brakenhoff R., Bradford C., Sorensen J., Rinaldo A., Rodrigo J. (2016). Moving towards personalised therapy in head and neck squamous cell carcinoma through analysis of next generation sequencing data. Eur. J. Cancer.

[46] Lawrence M.S., Stojanov P., Polak P., Kryukov G.V., Cibulskis K., Sivachenko A., Carter S.L., Stewart C., Mermel C.H., Roberts S.A. (2013). Mutational heterogeneity in cancer and the search for new cancer-associated genes. Nature.

[47] Rooney M.S., Shukla S.A., Wu C.J., Getz G., Hacohen N. (2015). Molecular and genetic properties of tumors associated with local immune cytolytic activity. Cell.

[48] Ren L., Leisegang M., Deng B., Matsuda T., Kiyotani K., Kato T., Harada M., Park J.-H., Saloura V., Seiwert T. (2019). Identification of neoantigen-specific T cells and their targets: Implications for immunotherapy of head and neck squamous cell carcinoma. OncoImmunology.

[49] Yang W., Lee K.-W., Srivastava R.M., Kuo F., Krishna C., Chowell D., Makarov V., Hoen D., Dalin M.G., Wexler L. (2019). Immunogenic neoantigens derived from gene fusions stimulate T cell responses. Nat. Med..

[50] Davidson E.J., A Davidson J., Sterling J.C., Baldwin P.J.W., Kitchener H.C., Stern P.L. (2003). Association between human leukocyte antigen polymorphism and human papillomavirus 16-positive vulval intraepithelial neoplasia in British women. Cancer Res..

[51] Qiu X., Zhang F., Chen D., Azad A.K., Zhang L., Yuan Y., Jiang Z., Liu W., Tan Y., Tao N. (2011). HLA-B*07 is a high risk allele for familial cervical cancer. Asian Pac. J. Cancer Prev..

[52] Hildesheim A., Schiffman M., Scott D.R., Marti D., Kissner T., E Sherman M., Glass A.G., Manos M.M., Lorincz A.T., Kurman R.J. (1998). Human leukocyte antigen class I/II alleles and development of human papillomavirus-related cervical neoplasia: Results from a case-control study conducted in the United States. Cancer Epidemiol. Biomark. Prev..

[53] Vita R., Mahajan S., A Overton J., Dhanda S.K., Martini S., Cantrell J.R., Wheeler D.K., Sette A., Peters B. (2019). The Immune Epitope Database (IEDB): 2018 update. Nucleic Acids Res..

[54] Chattopadhyay K. (2011). A comprehensive review on host genetic susceptibility to human papillomavirus infection and progression to cervical cancer. Indian J. Hum. Genet..

[55] Grandis J.R., Falkner D.M., Melhem M.F., E Gooding W., Drenning S.D., A Morel P. (2000). Human leukocyte antigen class I allelic and haplotype loss in squamous cell carcinoma of the head and neck: Clinical and immunogenetic consequences. Clin. Cancer Res..

[56] Nasman A., Andersson E., Nordfors C., Grün N., Johansson H., Munck-Wikland E., Massucci G., Dalianis T., Ramqvist T. (2013). MHC class I expression in HPV positive and negative tonsillar squamous cell carcinoma in correlation to clinical outcome. Int. J. Cancer.

[57] Girdlestone J., Isamat M., Gewert D., Milstein C. (1993). Transcriptional regulation of HLA-A and -B: Differential binding of members of the Rel and IRF families of transcription factors. Proc. Natl. Acad. Sci. USA.

[58] Meissner T.B., Liu Y.-J., Lee K.-H., Li A., Biswas A., Van Eggermond M.C.J.A., van den Elsen P.J., Kobayashi K.S. (2012). NLRC5 cooperates with the RFX transcription factor complex to induce MHC class I gene expression. J. Immunol..

[59] Gobin S.J., Keijsers V., Van Zutphen M., van den Elsen P.J. (1998). The role of enhancer A in the locus-specific transactivation of classical and nonclassical HLA class I genes by nuclear factor kappa B. J. Immunol..

[60] Ladasky J.J., Boyle S., Seth M., Li H., Pentcheva T., Abe F., Steinberg S.J., Edidin M. (2006). Bap31 enhances the endoplasmic reticulum export and quality control of human class I MHC molecules. J. Immunol..

[61] Hewitt E.W. (2003). The MHC class I antigen presentation pathway: Strategies for viral immune evasion. Immunology.

[62] Rizvi S.M., Del Cid N., Lybarger L., Raghavan M. (2011). Distinct functions for the glycans of tapasin and heavy chains in the assembly of MHC class I molecules. J. Immunol..

[63] Rizvi S.M., Raghavan M. (2010). Mechanisms of function of tapasin, a critical major histocompatibility complex class I assembly factor. Traffic.

[64] Fairfax B.P., Makino S., Radhakrishnan J., Plant K., Leslie S., Dilthey A., Ellis P., Langford C., Vannberg F.O., Knight J.C. (2012). Genetics of gene expression in primary immune cells identifies cell type-specific master regulators and roles of HLA alleles. Nat. Genet..

[65] Shukla S.A., Rooney M.S., Rajasagi M., Tiao G., Dixon P.M., Lawrence M.S., Stevens J., Lane W.J., DellaGatta J.L., Steelman S. (2015). Comprehensive analysis of cancer-associated somatic mutations in class I HLA genes. Nat. Biotechnol..

[66] Ludwig M.L., Kulkarni A., Birkeland A.C., Michmerhuizen N.L., Foltin S.K., Mann J.E., Hoesli R.C., Devenport S.N., Jewell B.M., Shuman A.G. (2018). The genomic landscape of UM-SCC oral cavity squamous cell carcinoma cell lines. Oral Oncol..

[67] Mann J.E., Kulkarni A., Birkeland A.C., Kafelghazal J., Eisenberg J., Jewell B.M., Ludwig M.L., Spector M.E., Jiang H., Carey T.E. (2019). The molecular landscape of the University of Michigan laryngeal squamous cell carcinoma cell line panel. Head Neck.

[68] Robinson J., Soormally A.R., Hayhurst J.D., Marsh S.G. (2016). The IPD-IMGT/HLA Database—New developments in reporting HLA variation. Hum. Immunol..

[69] Gensterblum-Miller E., Wu W., Sawalha A.H., Gensterblum E. (2018). Novel Transcriptional Activity and Extensive Allelic Imbalance in the Human MHC Region. J. Immunol..

[70] Hafner N., Driesch C., Gajda M., Jansen L., Kirchmayr R., Runnebaum I.B., Dürst M. (2008). Integration of the HPV16 genome does not invariably result in high levels of viral oncogene transcripts. Oncogene.

[71] Scheffner M., Werness B.A., Huibregtse J.M., Levine A.J., Howley P.M. (1990). The E6 oncoprotein encoded by human papillomavirus types 16 and 18 promotes the degradation of p53. Cell.

[72] van Houten V.M., Snijders P.J., van den Brekel M.W., Kummer J.A., Meijer C.J., van Leeuwen B., Denkers F., Smeele L.E., Snow G.B., Brakenhoff R.H. (2001). Biological evidence that human papillomaviruses are etiologically involved in a subgroup of head and neck squamous cell carcinomas. Int. J. Cancer.

[73] Dyson N., Guida P., Münger K., Harlow E. (1992). Homologous sequences in adenovirus E1A and human papillomavirus E7 proteins mediate interaction with the same set of cellular proteins. J. Virol..

[74] Heller C., Weisser T., Mueller-Schickert A., Rufer E., Hoh A., Leonhardt R.M., Knittler M.R. (2011). Identification of Key Amino Acid Residues That Determine the Ability of High Risk HPV16-E7 to Dysregulate Major Histocompatibility Complex Class I Expression. J. Boil. Chem..

[75] Georgopoulos N.T., Proffitt J.L., Blair G.E. (2000). Transcriptional regulation of the major histocompatibility complex (MHC) class I heavy chain, TAP1 and LMP2 genes by the human papillomavirus (HPV) type 6b, 16 and 18 E7 oncoproteins. Oncogene.

[76] Li H., Zhan T., Li C., Liu M., Wang Q.K. (2009). Repression of MHC class I transcription by HPV16E7 through interaction with a putative RXRbeta motif and NF-kappaB cytoplasmic sequestration. Biochem. Biophys. Res. Commun..

[77] Šímová J., Polláková V., Indrová M., Mikyšková R., Bieblová J., Štěpánek I., Bubeník J., Reinis M. (2011). Immunotherapy augments the effect of 5-azacytidine on HPV16-associated tumours with different MHC class I-expression status. Br. J. Cancer.

